# Supplementary figures and images for: Immune complex deposition promotes NK cell accumulation in the kidney
Source: PLoS One. 2024 Nov 21;19(11):e0312141. doi: 10.1371/journal.pone.0312141 (PMC11581347; doi:10.1371/journal.pone.0312141)

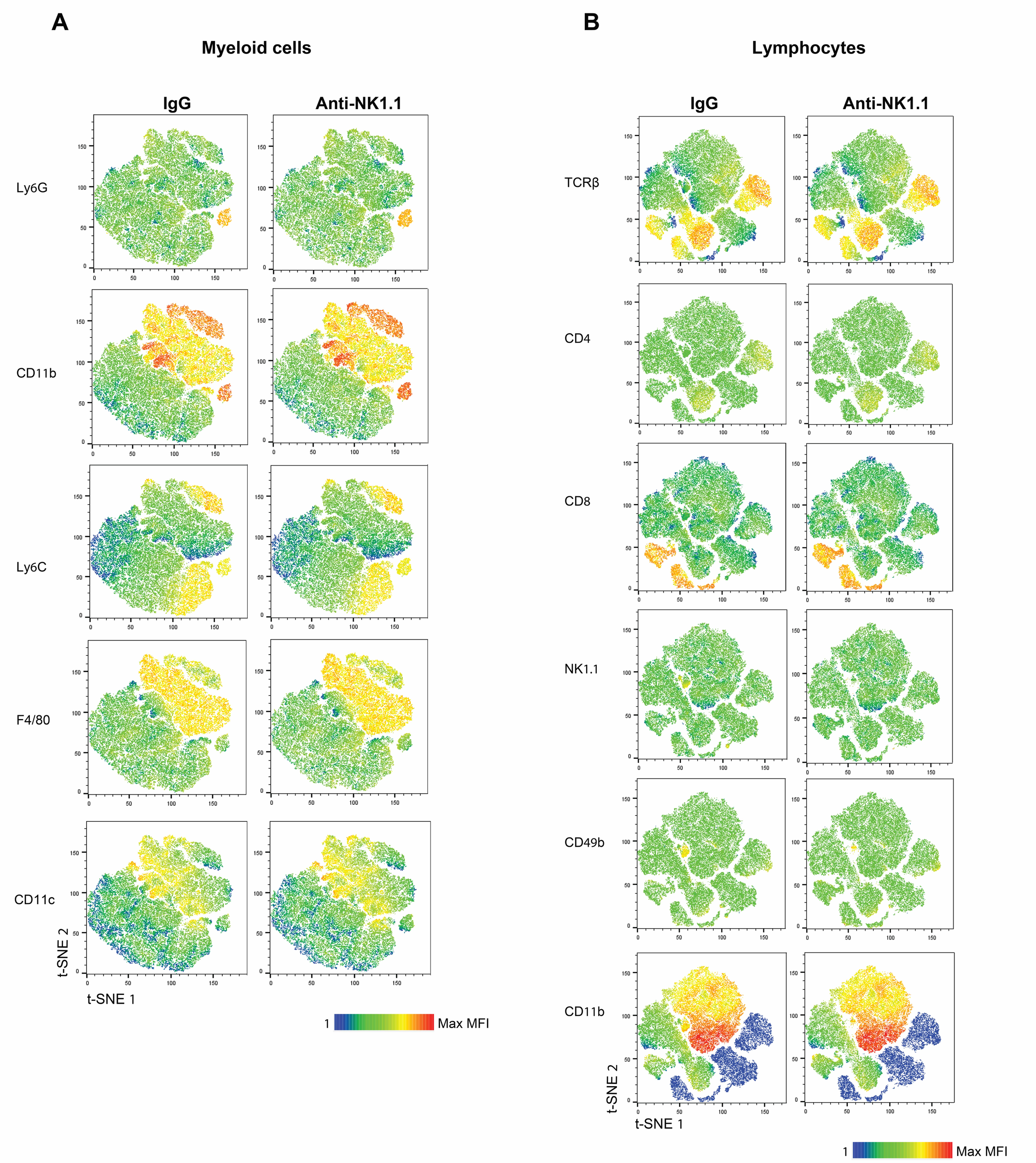

Supplement: S1 Fig — Flow cytometry was used to determine leukocyte populations in kidneys at day 7 after induction of the IC-mediated glomerulonephritis. Expression of the indicated (A) myeloid and (B) lymphoid markers on the CD45+ cells, is shown as heatmap in T-Distributed Stochastic Neighbor Embedding (tSNE) plots. (TIF) [file pone.0312141.s001.tif]

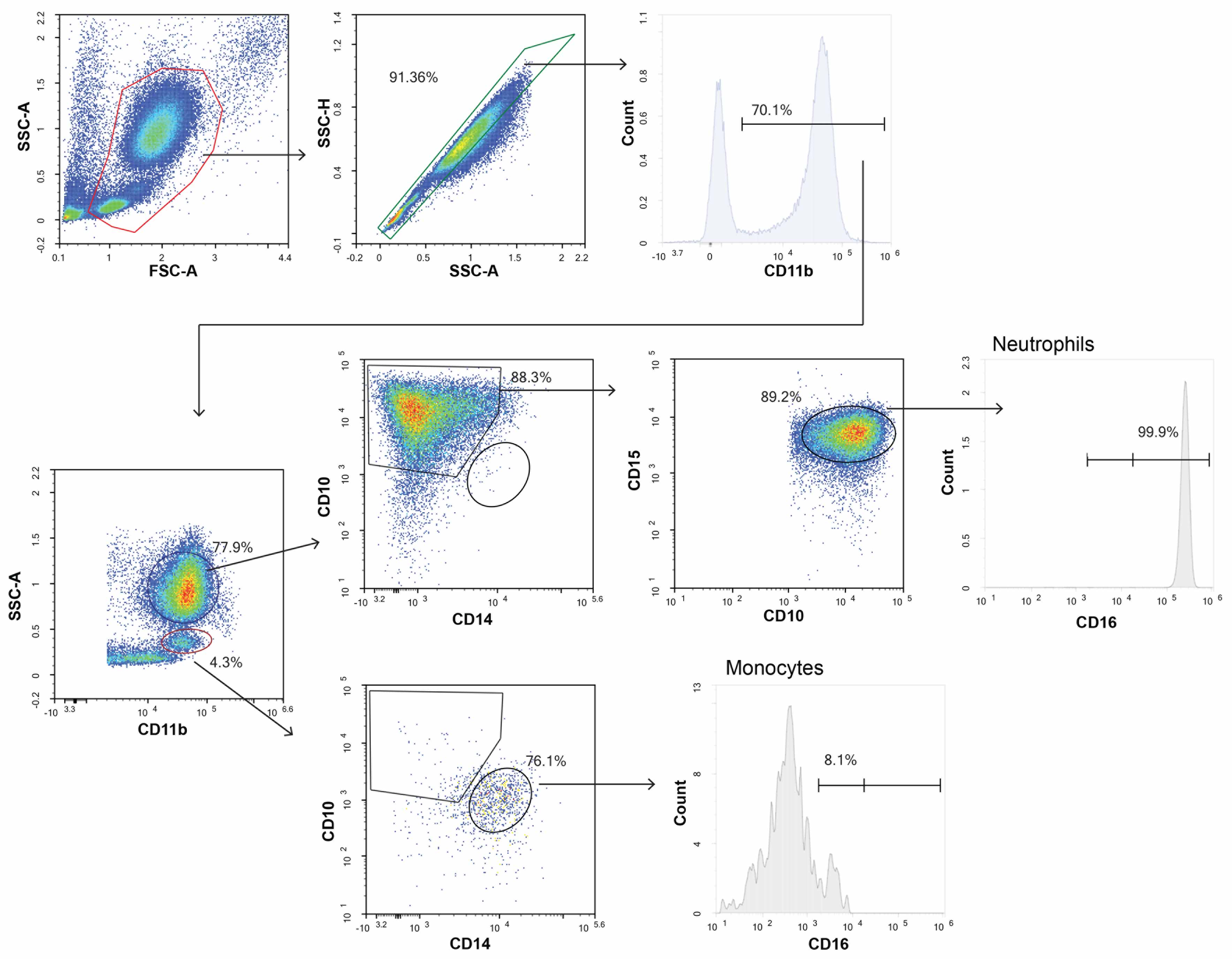

Supplement: S2 Fig — (TIF) [file pone.0312141.s002.tif]

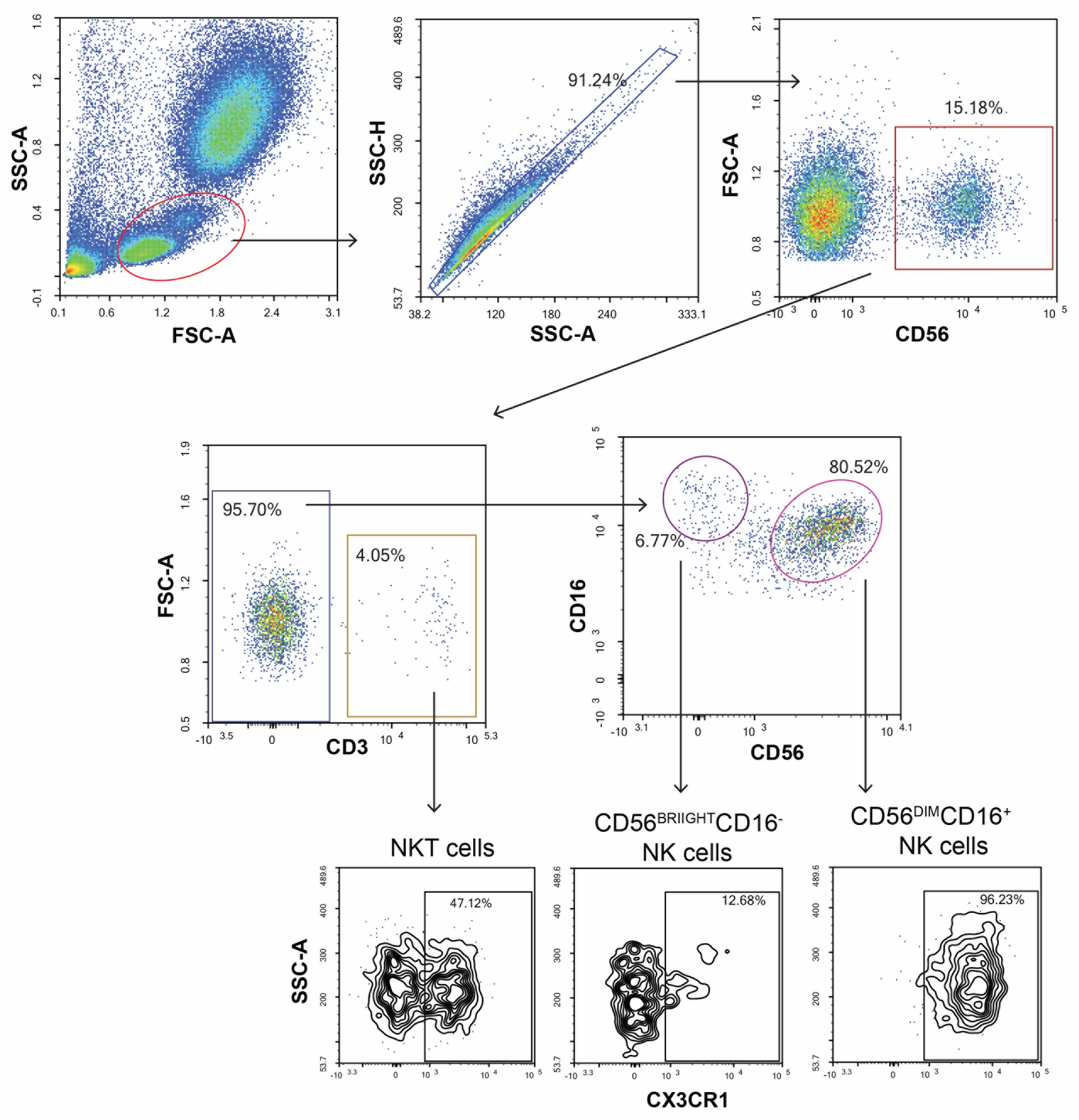

Supplement: S3 Fig — (TIF) [file pone.0312141.s003.tif]

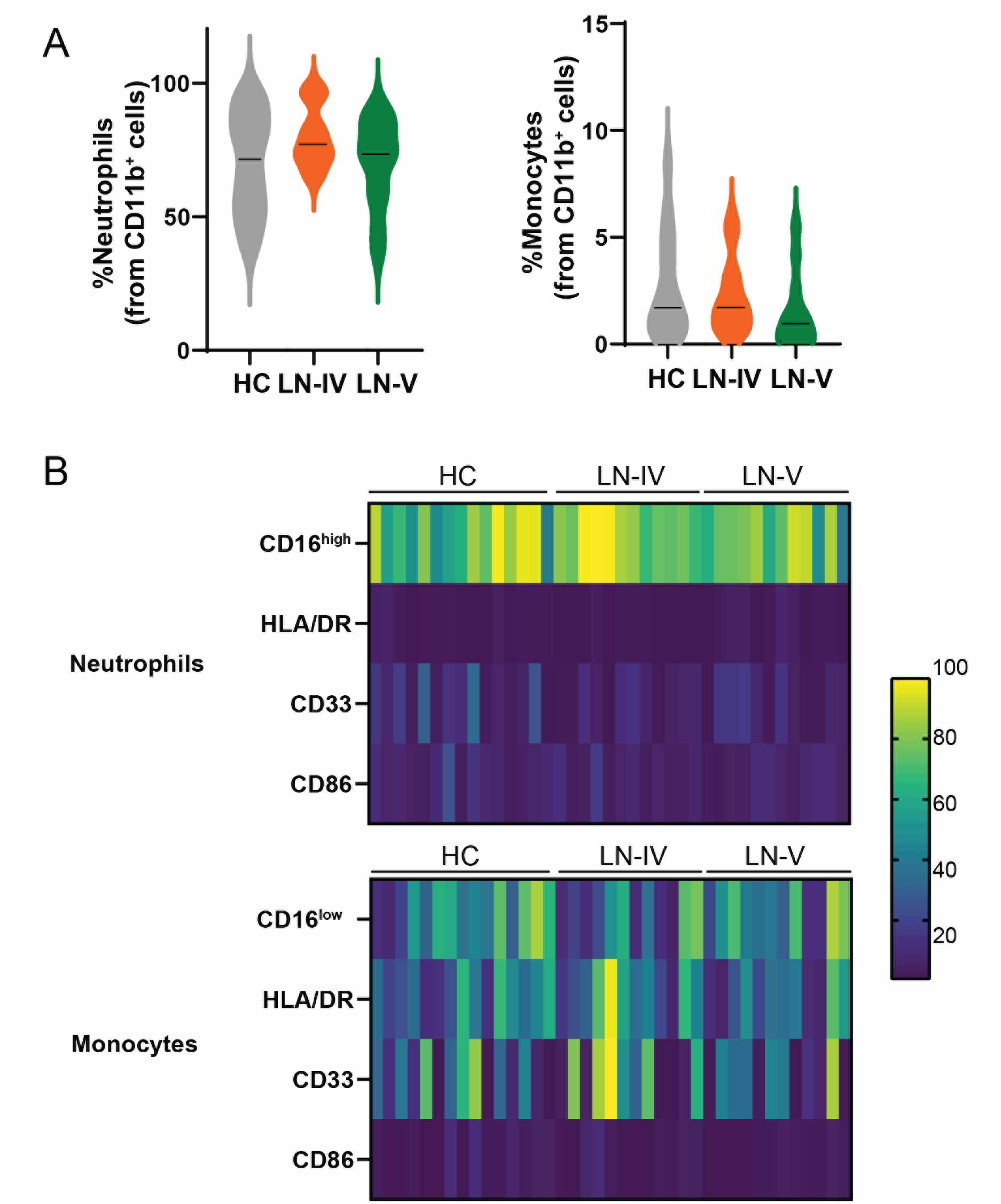

Supplement: S4 Fig — (A) Frequency of Neutrophil and Monocytes was evaluated by flow cytometry in peripheral blood of the indicated groups. (B) Heatmaps represent the proportions of the indicated activation markers analyzed by flow cytometry. (TIF) [file pone.0312141.s004.tif]
