# Supplementary material for: Immune complex deposition promotes NK cell accumulation in the kidney
Source: PLoS One. 2024 Nov 21;19(11):e0312141. doi: 10.1371/journal.pone.0312141 (PMC11581347; doi:10.1371/journal.pone.0312141)
Supplement: S1 Table — (DOCX) [file pone.0312141.s005.docx]

**S1 Supplemental Table.** Antibodies and reagents used in the study

| **Reagent** | **Brand** | **Cat #** |
| --- | --- | --- |
| Human anti-CD56 PE | TONBO | 50-0564-T100 |
| Human anti-CD11b APC-Cy7 | BioLegend | 301342 |
| Human anti-CD57 PE-CF594 | BD Horizon | 562488 |
| Human anti-CD16 PE-Cy7 | TONBO | 60-0166-T100 |
| Human anti-CXCR3 Alexa Fluor 488 | BioLegend | 353709 |
| Human anti-CX3CR1 APC | Invitrogen | 17-6099-42 |
| Human anti-CXCR4 BV605 | BioLegend | 306522 |
| Human anti-CD33 PerCP-Cy5.5 | BioLegend | 303414 |
| Human anti-CD15 FITC | BioLegend | 301903 |
| Human anti-HLA-DR AF700 | Invitrogen | 56-9956-42 |
| Human anti-CD11c violet Fluor 450 | TONBO | 75-0116-T100 |
| Human anti-CD11b PE | BioLegend | 301306 |
| Human anti-CD14 APC | BioLegend | 325608 |
| Human anti-CD86 BV650 | BioLegend | 305428 |
| Mouse anti-CD49b PE | TONBO | 50-0491-U100 |
| Mouse anti-NK1.1 APC-Fire750 | BioLegend | 108752 |
| Mouse anti-CD11b PE-Cy7 | BioLegend | 60-0112-U100 |
| Mouse anti-TCRβ FITC | TONBO | 35-5961-U500 |
| Mouse anti-CD4 PercCP-Cy5.5 | TONBO | 65-0041-U100 |
| Mouse anti-CD8 APC | TONBO | 20-0081-U100 |
| Mouse anti-CD45.2 VF450 | TONBO | 75-0454-U100 |
| Ghost Dye Violet 510 | TONBO | 13-0870-T100 |
| Mouse anti-CD45.2 APC-Cy7 | TONBO | 25-0454-U100 |
| Mouse anti-F4/80 VF-450 | TONBO | 75-4801-U100 |
| Mouse anti-CD11c APC | TONBO | 20-0114-U100 |
| Mouse anti-Ly6G PE | TONBO | 50-1276-U100 |
| Mouse anti-Ly6C FITC | BioLegend | 128006 |
| Human truStainFcX | BioLegend | 422302 |
| BD PharmLyse | BD | 555899 |
| Fc-Shield (Purified Mouse anti-CD16/CD32) | TONBO | 70-0161-M001 |
| DNAase | Sigma-Aldrich | 10104159001 |
| Colagenase IV | Thermo Fisher | 17104019 |
