# Supplementary material for: Immune complex deposition promotes NK cell accumulation in the kidney
Source: PLoS One. 2024 Nov 21;19(11):e0312141. doi: 10.1371/journal.pone.0312141 (PMC11581347; doi:10.1371/journal.pone.0312141)
Supplement: S2 Table — (DOCX) [file pone.0312141.s006.docx]

**S2 Supplemental Table.** Demographic characteristics of patients and healthy controls included in the study

| **Variable** | **Healthy Controls**  **(n=14)** | **Type IV LN patients**  **(n=16)** | **Type V LN patients**  **(n=14)** | ***P* value** |
| --- | --- | --- | --- | --- |
| Age at evaluation, yr | 33.2 ± 12.2 | 35.6 ± 8.7 | 38.6 ± 11.7 | 0.4375 |
| Female n, (%) | 12 (85.7%) | 14 (87.5%) | 13 (92.9%) | 0.3347 |
|  |  |  |  |  |
| Age at diagnosis SLE, yr  (mean ± SD) |  | 21.7 ± 6.5 | 27.4 ± 8.5 | *0.0487* |
| Age at diagnosis LN, yr  (mean ± SD) |  | 23.9 ± 8.3 | 21.4 ± 31.9 | 0.7638 |
|  |  |  |  |  |
| **Disease duration** |  |  |  |  |
| SLE (mean ± SD) |  | 14.5 ± 4.6 | 11.7 ± 6.0 | 0.1626 |
| LN (mean ± SD) |  | 12.3 ± 4.0 | 9.5 ± 5.9 | 0.1565 |
|  |  |  |  |  |
| SLEDAI |  |  |  |  |
| Baseline (mean ± SD) |  | 14.1 ± 6.6 | 13.8 ± 5.9 | 0.8843 |
| 24 months (mean ± SD) |  | 5.7 ± 3.4 | 4.7 ± 4.8 | 0.5099 |
| 36 months (mean ± SD) |  | 7.3 ± 4.7 | 7.6 ± 4.5 | 0.8889 |
