# Supplementary material for: Immune complex deposition promotes NK cell accumulation in the kidney
Source: PLoS One. 2024 Nov 21;19(11):e0312141. doi: 10.1371/journal.pone.0312141 (PMC11581347; doi:10.1371/journal.pone.0312141)
Supplement: Attachment — Submitted filename: Response to Reviewers.docx [file pone.0312141.s008.docx]

We would like to thank both reviewers for the time and effort devoted to the review of this manuscript. We answered all their points and believe that the manuscript has improved substantially.

Reviewer #1

1. Do you have histological analysis of renal tissue from mouse at Day 7 when the mitigating effect of NK depletion is observed, rather than at day 21 when the effect on proteinuria has disappeared?

We did evaluate the renal histology at day 7. However, we observed very modest changes (histological score 1.7 ± 0.2 *vs* 1.4 ± 0.24; p=0.34). We decided to evaluate day 21, reasoning that tissue changes might be slower to show than changes in proteinuria. Further, we thought that the information from day 21 would better reflect how the initial inflammation might impact the establishment of the disease, and whether changes during the initial time points were enough to modify histology. Unfortunately, this was not the case, perhaps because the magnitude of the initial perturbance was modest. We have included the histological information of day 7 in the Results, to strengthen this point. Thank you for the suggestion.

2. What is the phenotype of the residual NK cells seen in renal material after NK depletion (Fig 1G)? Are these NK cells CD56 bright or dim, are they expressing activation markers?

Murine NK cells lack CD56 or an analogous molecule. Therefore, we evaluated CD11b as an activation marker. At day 7 after disease induction, no differences in CD11b expression on NK cells were observed. However, at day 21 after disease induction, the few remaining NK cells in NK-cell depleted animals showed a tendency to have higher expression levels of CD11b (MFI: 6544 ± 737 vs. 10884 ± 1864; p=0.07) compared to the NK cells from mice that received the control antibody. This information, and its potential implications in the course of the disease, has been included in the Results and Discussion section.

3. Can you confirm that the correlation of SLEDAI with CD56dim NK cells is with SLEDAI scored at diagnosis rather than at the time of when the sample (for flow cytometry) was taken or are these the same time point?

Yes, the correlation was done with the SLEDAI at the time of diagnosis. We also evaluated if it correlated with the SLEDAI at the moment of the sample acquisition, however, the disease activity of most patients was very low at the time of sample acquisition.

4. The resolution of the Figures is not great.

We have improved the quality of the uploaded files.

Reviewer #2:

1. Discuss the potential future impact of these findings on the prevention of damage, e.g. through modulation of NK cell responses.

A more extensive analysis of the possible impact of these findings has been added to the Discussion section.

2. The authors should clarify further (maybe in the methods) what their contribution to figure 1 was. Did they access data and (re-)analyse?

Fig 1A is data directly obtained from the SLE-AMP Consortium website. A more clear explanation has been included in the Methods section.

3. What was the sex distribution among healthy controls? Because the number of CD56bright NK cells within peripheral blood decline with increasing age and are more common stable in women, this is important information (doi: 10.1186/1742-4933-3-10; <https://doi.org/10.1016/j.mad.2016.04.001>). Were SLE patients more commonly female when compared to controls? If so, the authors may even under-estimate differences between groups?

Controls were sex and aged matched. We have included that info in S2 Table. Additionally, a statement of the importance of this issue has been included in the Results section, as well as the reference that supports it.

4. In Figure 2A the authors claim a tendency towards altered NK cell subsets within patients versus controls. The differences appear minor, and it would be helpful to know the p value to assess the statement. In Figure 2C, can the authors quantify their statement that CD11 differs between groups, and a legend to the heat map would be helpful (colour gradient represents expression levels or p values?).

Yes, we agree with the reviewer that the differences are minor. We have included the p values of the corresponding analysis as well as the missing legend for the colorimetric gradient in order to be more accurate.

5. This referee recommends considering this manuscript as a “short article” after addressing minor points outlined above.

We agree that this work would correspond to a “brief communication” or a “short article”, however, we believe PlosOne does not have that type of article format.
